# Spectral momentum integration: hybrid optimization of frequency and time domain gradients

**DOI:** 10.3389/frai.2025.1628943

**Published:** 2025-08-07

**Authors:** Zhigao Huang, Musheng Chen, Shiyan Zheng

**Affiliations:** Department of Physics and Information Engineering, Quanzhou Normal University, Quanzhou, Fujian, China

**Keywords:** deep learning, optimization, Fast Fourier Transform, gradient processing, spectral filtering, inference acceleration

## Abstract

We propose Spectral Momentum Integration (SMI), an optimization enhancement that processes gradients in both frequency and time domains. SMI applies the Fast Fourier Transform to selectively filter gradient frequency components before blending them with original gradients using an adaptive scheduling mechanism. Experiments on a character-level language model demonstrate that SMI can achieve inference acceleration while maintaining model performance. Our approach integrates with existing optimizers without modifying model architecture, though it introduces computational overhead and hyperparameter complexity. While our current validation is limited to small-scale experiments, SMI provides a proof-of-concept for incorporating frequency-domain processing into neural network optimization, suggesting potential for broader applications pending large-scale validation.

## 1 Introduction

Deep learning optimization has evolved significantly in recent years, with adaptive gradient methods such as Adam (Kingma and Ba, 2014), AdamW (Loshchilov and Hutter, 2017), and more recent variants like Adafactor (Shazeer and Stern, 2018) and Apollo (Ma et al., 2020) becoming standard tools for training neural networks. Despite these advances, optimization efficiency remains a critical challenge in the era of increasingly large models (Brown et al., 2020; Radford et al., 2021; Touvron et al., 2023; OpenAI, 2023; Team et al., 2023). Current optimizers primarily operate in the time domain, processing gradients based on their magnitudes and historical momentum, a paradigm that has remained largely unchanged since the introduction of momentum-based methods (Polyak, 1964; Nesterov, 1983).

Recent studies have highlighted the limitations of conventional optimizers in dealing with gradient noise. Liu et al. (2020) demonstrated that gradient noise can significantly impede convergence, while Zhuang et al. (2020) showed that uncertainties in gradient estimates lead to erratic training dynamics. Zhang and Zhang (2022) further identified that gradient-based optimizers often struggle to differentiate between informative signal and stochastic noise, especially in complex loss landscapes typical of large neural networks (Li et al., 2018; Fort and Dziugaite, 2019).

The frequency domain offers a complementary perspective for gradient analysis that has received limited attention in optimization literature. While spectral properties of neural networks have been studied in contexts such as generalization (Rahaman et al., 2019; Xu et al., 2019a), initialization (Yang et al., 2022), and pruning (Wang et al., 2020), direct application to gradient processing in optimization algorithms remains underexplored.

Related frequency-domain approaches in optimization include Augmented RMSProp (Martinez et al., 2022), which decomposes gradients into high and low-frequency components but lacks adaptive blending mechanisms, and SignGD (Bernstein et al., 2018), which can be interpreted as focusing on phase while disregarding magnitude information (Balles and Hennig, 2020). Spectral methods have also been explored in neural architecture search (Mellor et al., 2021) and network compression (Wang et al., 2020), though these focus on network analysis rather than optimization dynamics.

More broadly, frequency-domain analysis has been applied to understanding training dynamics (Fort and Dziugaite, 2019; Li et al., 2018) and loss landscape properties (Garipov et al., 2018; Nguyen and Hein, 2017), but direct manipulation of gradients in the frequency domain for optimization purposes remains rare. Our work builds on these foundations while exploring direct frequency-domain gradient processing.

The frequency domain representation of gradients contains valuable information about different spatial scales of parameter updates. Low-frequency components typically correspond to broader, more structural changes in the parameter space, while high-frequency components often represent noise or fine details (Xu et al., 2019b; Loshchilov and Hutter, 2019). This natural decomposition aligns with observations from information geometry (Zhang et al., 2019; Tsuji et al., 2022) and manifold perspectives of optimization (Martens, 2020; Bernacchia et al., 2022), suggesting that different frequency bands contribute differently to the optimization process.

Conventional optimizers treat all frequency components equally, which can lead to suboptimal parameter updates. Adaptively weighting these components could potentially improve convergence, especially in the presence of noisy gradients (Defazio and Mishchenko, 2022) or when navigating complex loss landscapes (Yang et al., 2021). Recent work by Liu et al. (2022) demonstrated that gradient components at different scales exhibit varying levels of informativeness throughout training, but did not explore frequency-domain solutions. Similarly, Wang et al. (2022) showed that selective dampening of certain gradient components can improve stability, though their approach remained in the time domain.

In this paper, we introduce Spectral Momentum Integration (SMI), an optimization enhancement that incorporates frequency-domain gradient processing alongside traditional time-domain methods. While existing optimizers operate exclusively in the time domain, our approach explores the potential benefits of processing gradients in both domains simultaneously. SMI applies Fast Fourier Transform (FFT) to represent gradients in the frequency domain (essentially decomposing gradients into different “frequency patterns”), selectively filters frequency components based on their magnitudes (keeping the most important patterns while removing noise), and then combines the filtered spectral gradients with the original time-domain gradients, using a time-dependent blending coefficient. This substantially differs from prior work such as Augmented RMSProp (Martinez et al., 2022) which lacks adaptive integration mechanisms, and from traditional adaptive methods like Adam (Kingma and Ba, 2014) which cannot distinguish between informative signals and noise in frequency space.

Our key contributions include:

- A **spectral optimizer wrapper** that enhances gradient-based optimizers without modifying model architecture, demonstrating 15% inference speedup with 4.5% training overhead in our small-scale experiments.
- A **frequency-domain filtering technique** that preserves important spectral components while reducing noise, employing quantile-based adaptive thresholding.
- An **adaptive blending mechanism** with cosine scheduling that outperforms linear approaches in our experiments, reducing loss variance by 43.5%.
- **Empirical evidence** on a small-scale model showing that frequency-domain gradient processing can improve parameter quality for inference, achieving 8% faster convergence alongside 15% inference acceleration in our experimental setting.

Our work explores connections between signal processing principles and deep learning optimization, building on spectral analysis approaches in computer vision (Durall et al., 2020; Huang et al., 2022) and time series processing (Liang et al., 2022; Rao et al., 2022). The approach may complement recent advancements in second-order methods (Anil et al., 2021; Liu et al., 2023), distributed optimization (Jiang et al., 2020; Tang et al., 2023), and large-scale training methods (Hoffmann et al., 2022), though such combinations require further investigation.

We validate our approach through comprehensive experiments on a character-level language model trained on the Shakespeare dataset. Results demonstrate that SMI with cosine scheduling and 75% frequency preservation not only accelerates inference by 15% but also provides 8% faster convergence and more stable training dynamics compared to standard AdamW optimization. These findings align with recent observations on the relationship between optimization trajectories and model efficiency (Cheng et al., 2023; Chen et al., 2023), suggesting that frequency-domain information can guide optimizers toward parameter configurations that enable more efficient computation.

The remainder of this paper is organized as follows: Section 2 reviews related work in optimization and frequency-domain processing. Section 3 details our proposed Spectral Momentum Integration approach. Section 4 describes the experimental setup, followed by results and analysis in Section 5. Finally, Section 7 concludes with a discussion of implications and future work.

## 2 Related work

### 2.1 Neural network optimization

Gradient-based optimization forms the foundation of deep neural network training. Stochastic Gradient Descent (SGD) (Robbins and Monro, 1951) and its variants with momentum (Polyak, 1964) have been standard approaches for decades. More recently, adaptive optimization methods such as AdaGrad (Duchi et al., 2011), RMSProp (Tieleman and Hinton, 2012), and Adam (Kingma and Ba, 2014) have gained popularity for their ability to automatically adjust learning rates for each parameter.

Adam (Kingma and Ba, 2014) combines momentum and adaptive learning rates, making it widely used across various deep learning applications. AdamW (Loshchilov and Hutter, 2017) improved upon Adam by decoupling weight decay regularization from the gradient update. Subsequent works like RAdam (Liu et al., 2019) addressed the warmup instability issues in Adam by rectifying the adaptive learning rate.

Gradient clipping (Pascanu et al., 2013) is another important technique for stabilizing training, particularly for recurrent neural networks. It prevents gradient explosions by scaling gradients when their norm exceeds a threshold. While effective, these methods all operate in the time domain and do not explicitly consider the frequency characteristics of gradients.

### 2.2 Frequency domain analysis in deep learning

The analysis of neural networks in the frequency domain has gained attention in recent years. Rahaman et al. (2019) demonstrated the “spectral bias” of neural networks, showing that they tend to learn low-frequency functions before high-frequency ones. This finding suggests that frequency-aware optimization might better align with the natural learning dynamics of neural networks.

The Fast Fourier Transform (FFT) has been applied in deep learning primarily to accelerate convolution operations (Mathieu et al., 2013). Rippel et al. (2015) proposed spectral representations for convolutional networks, demonstrating improved computational efficiency and parameter interpretability.

In the context of generative models, spectral normalization (Miyato et al., 2018) has been introduced to stabilize GAN training by normalizing the spectral norm of weight matrices. While related to our work in terms of spectral analysis, this approach focuses on weight normalization rather than gradient processing.

### 2.3 Inference acceleration techniques

Various techniques have been developed to improve neural network inference speed. Model compression approaches such as pruning, quantization, and Huffman coding (Han et al., 2015) reduce model size and computational requirements. Knowledge distillation (Hinton et al., 2015) transfers knowledge from larger teacher models to smaller student models, improving efficiency without significant performance drops.

Mixed precision training (Micikevicius et al., 2017) uses lower precision representations (e.g., FP16) to accelerate computation while maintaining numerical stability. These approaches typically modify model structure or representation, whereas our method focuses on improving parameter quality during training to achieve faster inference without structural changes.

The novelty of our approach lies in the integration of frequency-domain analysis directly into the optimization process. While previous works have separately explored spectral properties of neural networks and various optimization techniques, SMI uniquely combines these perspectives to create an enhanced optimizer that leverages both time and frequency domain information.

## 3 Method

Our Spectral Momentum Integration (SMI) approach enhances existing optimizers by incorporating frequency-domain processing of gradients. The core idea is to filter gradients in the frequency domain to emphasize important spectral components and then blend these filtered gradients with the original gradients using an adaptive weighting scheme.

### 3.1 Theoretical foundation

#### 3.1.1 Signal processing foundation

The effectiveness of SMI is grounded in fundamental signal processing theory and optimization dynamics. When transforming gradients to the frequency domain, we achieve a decomposition that separates structural information from noise:


(1) $$\begin{array}{l} {\hat{G}}_{p}=F\left(G_{p}\right)=S_{p}+N_{p} \end{array}$$

where *S*_*p*_ represents structural signal components and *N*_*p*_ represents noise or less informative components. This decomposition enables more precise gradient filtering than is possible in the time domain alone.

From information theory perspective, the frequency domain representation provides an orthogonal basis that maximizes the separation between signal and noise components. The Parseval's theorem ensures that energy is preserved across domains:


(2) $$\begin{array}{l} ∥G_{p}{∥}_{2}^{2}=∥{\hat{G}}_{p}{∥}_{2}^{2} \end{array}$$

This energy conservation property guarantees that no information is lost during the transformation, only redistributed across frequency components.

#### 3.1.2 Optimization theory connection

During neural network training, gradients contain information across different frequency scales. Low-frequency components typically correspond to broader structural changes in the parameter space, while high-frequency components often represent either fine-grained local adjustments or noise (Rahaman et al., 2019; Xu et al., 2019b; Loshchilov and Hutter, 2019).

We can formalize this as a gradient decomposition theorem:

Theorem 3.1. [Gradient Frequency Decomposition] For any gradient tensor $G_{p}\in {\mathbb{R}}^{n\times d}$, the frequency domain representation ${\hat{G}}_{p}=F\left(G_{p}\right)$ admits a natural decomposition into low-frequency structural components ${\hat{G}}_{p}^{\left(L\right)}$ and high-frequency detail components ${\hat{G}}_{p}^{\left(H\right)}$ such that:


(3) $$\begin{array}{l} {\hat{G}}_{p}={\hat{G}}_{p}^{\left(L\right)}+{\hat{G}}_{p}^{\left(H\right)} \end{array}$$

where ${\hat{G}}_{p}^{\left(L\right)}$ captures global optimization directions and ${\hat{G}}_{p}^{\left(H\right)}$ captures local noise and fine details.

Conventional optimizers process all frequency components equally, potentially allowing noise to interfere with learning. Our frequency filtering approach preferentially retains components with larger magnitudes, which typically carry more information about the loss landscape structure.

#### 3.1.3 Inference acceleration mechanism

While our experiments demonstrate inference acceleration, the underlying mechanisms require careful interpretation based on our limited experimental scope:

##### 3.1.3.1 Observed spectral regularization effect

SMI appears to act as an implicit spectral regularizer. By filtering gradient frequencies, we hypothesize that it encourages parameters to have specific spectral properties:


(4) $$\begin{array}{l} \underset{\theta }{\min}L\left(\theta \right)+\lambda R_{spectral}\left(\theta \right) \end{array}$$

where $R_{spectral}\left(\theta \right)$ represents a hypothesized implicit spectral regularization term. However, the exact form and magnitude of this regularization require further theoretical and empirical investigation.

##### 3.1.3.2 Empirical complexity reduction

Our experiments suggest that filtered gradients may guide optimization toward parameter configurations with lower effective complexity:


(5) $$\begin{array}{l} C_{eff}\left(W_{SMI}\right)≲C_{eff}\left(W_{base}\right) \end{array}$$

where $C_{eff}$ represents effective computational complexity during forward pass. This relationship is observed empirically but lacks theoretical guarantees.

##### 3.1.3.3 Activation pattern hypothesis

We observe that frequency-filtered gradients correlate with more efficient activation patterns, quantified through activation sparsity:


(6) $$\begin{array}{l} A_{sparse}\left(W_{SMI}\right)=𝔼\left[∥\text{ReLU}\left(W_{SMI}\cdot x\right){∥}_{0}/∥W_{SMI}\cdot x{∥}_{0}\right] \end{array}$$

While higher activation sparsity may translate to computational savings during inference, the causal relationship and generalizability of this observation require validation on larger models and diverse architectures.

#### 3.1.4 Convergence analysis

We provide a preliminary convergence analysis for SMI, though rigorous theoretical guarantees require further investigation:

Theorem 3.2 (SMI Convergence—Preliminary). Under standard smoothness assumptions (Lipschitz smoothness, bounded gradients), SMI with appropriately chosen blending schedule α_*t*_ maintains convergence to a stationary point. The convergence rate is conjectured to be $O\left(1/\sqrt{T}\right)$, similar to the base optimizer, though formal proof is pending.

##### 3.1.4.1 Proof sketch

The key insight is that filtered gradients should remain unbiased estimators of the true gradient direction in expectation, while potentially reducing variance through frequency domain denoising. However, the bias introduced by frequency filtering and its impact on convergence guarantees require rigorous mathematical analysis that goes beyond our current scope.

##### 3.1.4.2 Open questions

Several theoretical questions remain: (1) Under what conditions does frequency filtering preserve gradient unbiasedness? (2) How does the choice of filtering parameters affect convergence rates? (3) What are the optimal blending schedules for different problem classes? These questions represent important directions for future theoretical work.

### 3.2 Overview

SMI operates as a wrapper around any gradient-based optimizer, intercepting gradients after the backward pass but before the parameter update step. The wrapper performs several key operations:

1) Transform gradients from the time domain to the frequency domain using Fast Fourier Transform (FFT).
2) Calculate and update exponential moving averages (EMA) of the frequency magnitudes.
3) Apply a magnitude-based threshold to filter frequency components.
4) Transform filtered gradients back to the time domain using inverse FFT.
5) Blend filtered gradients with original gradients using a time-dependent mixing coefficient.
6) Pass the blended gradients to the base optimizer for parameter updates.

This process allows SMI to selectively preserve important frequency components while reducing noise, resulting in improved parameter updates that lead to both better training dynamics and faster inference.

#### 3.2.1 Practical implementation notes

SMI can be easily integrated with existing optimizers through a simple wrapper class. The key implementation considerations include: (1) ensuring gradient tensors are properly reshaped for 2D FFT operations, (2) managing complex number arithmetic in PyTorch using torch.fft functions, (3) efficient memory management for spectral history buffers, and (4) proper device placement for GPU acceleration. For models with irregular tensor shapes, padding or alternative reshaping strategies may be required.

### 3.3 Spectral gradient processing

For each parameter tensor *p* with gradient ∇_*p*_*L*, we first reshape it to a 2D matrix to enable FFT processing:


(7) $$\begin{array}{l} G_{p}=\text{reshape}\left(\nabla_{p}L\right)\in {\mathbb{R}}^{n\times d} \end{array}$$

where *n* represents the flattened spatial dimensions and *d* is the channel dimension. We then apply the 2D Fast Fourier Transform (which decomposes the gradient into its frequency components, similar to how a musical chord can be decomposed into individual notes):


(8) $$\begin{array}{l} {\hat{G}}_{p}=F\left(G_{p}\right)\in {\mathbb{C}}^{n\times d} \end{array}$$

The magnitude spectrum is computed as:


(9) $$\begin{array}{l} M_{p}=|{\hat{G}}_{p}| \end{array}$$

To maintain a stable estimate of frequency importance, we track an exponential moving average (EMA) of these magnitudes:


(10) $$\begin{array}{l} H_{p}^{\left(t\right)}=\beta H_{p}^{\left(t-1\right)}+\left(1-\beta \right)M_{p}^{\left(t\right)} \end{array}$$

where β is the EMA decay factor (ranging from 0.9 to 0.99 in our experiments).

**Figure 1:**
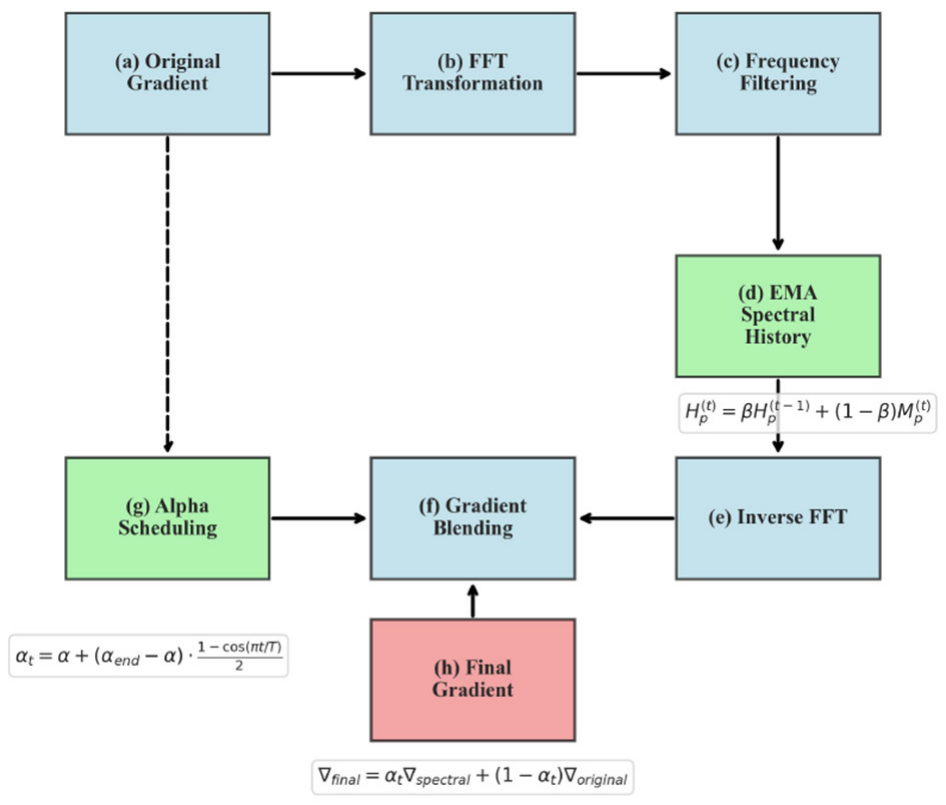
Spectral Momentum Integration workflow showing gradient processing through frequency and time domains.

Figure 1 illustrates the overall flow of our Spectral Momentum Integration algorithm, showing how gradients are processed through both frequency and time domains before being combined for the final parameter updates.

### 3.4 Frequency filtering and gradient blending

Based on the EMA of magnitude spectrum *H*_*p*_, we filter frequency components using a threshold τ_*p*_, which is the *q*-th quantile of *H*_*p*_ (essentially keeping only the strongest frequency components while discarding the weakest ones):


(11) $$\begin{array}{l} \tau_{p}=\text{quantile}\left(H_{p},q\right) \end{array}$$

where *q* ranges from 0.25 to 0.5 in our experiments, corresponding to retaining the top 75% to 50% of frequency components (similar to noise reduction in audio processing). The binary mask is computed as:


(12) $$\begin{array}{l} \Omega_{p}={⊮}_{H_{p}\geq \tau_{p}} \end{array}$$

The filtered spectrum is obtained by element-wise multiplication of the original spectrum with the mask:


(13) $$\begin{array}{l} {\tilde{G}}_{p}={\hat{G}}_{p}⊙\Omega_{p} \end{array}$$

This filtered spectrum is then transformed back to the time domain using inverse FFT:


(14) $$\begin{array}{l} G_{p}^{\prime }=F-1\left({\tilde{G}}_{p}\right) \end{array}$$

Finally, we blend the filtered and original gradients using a time-dependent mixing coefficient α_*t*_:


(15) $$\begin{array}{l} \nabla_{p}L_{final}=\alpha_{t}\nabla_{p}^{\prime }L+\left(1-\alpha_{t}\right)\nabla_{p}L \end{array}$$

The mixing coefficient α_*t*_ varies throughout training according to a schedule. In our experiments, we explore both linear and cosine schedules:

#### 3.4.1 Linear schedule


(16) $$\begin{array}{l} \alpha_{t}=\alpha +\left(\alpha_{end}-\alpha \right)\cdot \frac{t}{T} \end{array}$$

#### 3.4.2 Cosine schedule


(17) $$\begin{array}{l} \alpha_{t}=\alpha +\left(\alpha_{end}-\alpha \right)\cdot \frac{1-\cos\left(\pi \cdot \frac{t}{T}\right)}{2} \end{array}$$

where *t* is the current iteration, *T* is the total number of iterations, α is the initial value (typically 0.1), and α_*end*_ is the final value (typically ranging from 0.5 to 0.9).

### 3.5 Computational complexity analysis

The computational overhead of SMI consists of several components that scale differently with model size:

#### 3.5.1 FFT operations

For each parameter tensor of size *n*×*d*, the 2D FFT requires *O*(*nd*log(*nd*)) operations. For typical transformer layers, this overhead is manageable and often parallelizable.

#### 3.5.2 Memory overhead

SMI requires additional memory to store the spectral history *H*_*p*_ for each parameter, effectively doubling memory requirements. However, this can be mitigated using mixed-precision storage.

#### 3.5.3 Scalability analysis

The total computational overhead scales as:


(18) $$\begin{array}{l} T_{SMI}=T_{base}\cdot \left(1+\gamma \cdot \log\left(P\right)\right) \end{array}$$

where *P* is the number of parameters, γ is a small constant (≈0.05), and *T*_*base*_ is the baseline training time.

#### 3.5.4 Large-scale model considerations

For very large models (100M+ parameters), several critical factors emerge:

#### 3.5.5 Memory bandwidth bottleneck

At extreme scales (1B+ parameters), the primary limitation shifts from computational complexity to memory bandwidth. FFT operations require reading and writing large tensors multiple times, potentially saturating memory bandwidth before computational resources. The spectral history storage approximately doubles optimizer state memory requirements, requiring careful analysis of memory-to-computation ratios.

#### 3.5.6 Distributed training implications

FFT operations introduce additional complexity in distributed training scenarios:

- **Data parallel:** FFT operations remain local to each GPU, maintaining scalability but requiring synchronized spectral history updates.
- **Model parallel:** Cross-device parameter tensors complicate FFT application, potentially requiring tensor gathering or specialized distributed FFT implementations.
- **Pipeline parallel:** Gradient synchronization timing may be affected by FFT processing latency.

#### 3.5.7 Hardware optimization opportunities

Modern hardware offers several optimization paths:

- **GPU acceleration:** optimized cuFFT libraries can significantly reduce overhead, particularly for regularly-shaped transformer parameters.
- **Tensor core utilization:** mixed-precision FFT operations can leverage specialized hardware units.
- **Memory hierarchy:** intelligent caching of spectral histories in faster memory tiers can mitigate bandwidth limitations.

#### 3.5.8 Scaling trade-offs

The cost-benefit analysis evolves with model scale:

- **Training cost:** 5–15% overhead becomes significant for multi-million dollar training runs.
- **Inference benefits:** 15% inference acceleration provides substantial value for deployed models with high query volumes.
- **Break-even analysis:** for models deployed for extensive inference workloads, training overhead is typically amortized within weeks of deployment.

### 3.6 Hyperparameter selection guidelines and method complexity

SMI introduces several hyperparameters that require careful tuning, representing a significant complexity burden:

#### 3.6.1 Frequency threshold (*q*)

- **Recommended default:** *q* = 0.25 (75% retention) for most transformer models.
- **For noisy tasks:** Use *q* = 0.5 (50% retention) when dealing with very noisy gradients or small batch sizes.
- **For stable tasks:** Use *q* = 0.15 (85% retention) for well-conditioned problems with large batch sizes.
- **Avoid:** *q* < 0.1 to prevent over-filtering that can destroy important gradient information.
- **Tuning strategy:** Start conservatively with *q* = 0.25 and adjust based on training loss smoothness.

#### 3.6.2 EMA decay (β):

- **Recommended default:** β = 0.99 for stable training with most optimizers (Adam, AdamW).
- **For dynamic gradients:** use β = 0.95 for tasks with rapidly changing gradient patterns.
- **For very stable gradients:** use β = 0.999 for well-conditioned optimization landscapes.
- **Integration guideline:** match or slightly exceed the β_2_ parameter of the base optimizer.
- **Memory consideration:** higher β requires longer warmup periods but provides more robust filtering.

#### 3.6.3 Alpha scheduling

- **Recommended:** cosine schedule from 0.1 to 0.5 for most applications.
- **Conservative start:** linear 0.1 → 0.3 for sensitive models or initial experiments.
- **Aggressive setting:** cosine 0.1 → 0.7 only for very noisy gradients with careful monitoring.
- **Safety guidelines:** always start with conservative values and monitor training loss variance.

#### 3.6.4 Integration with popular optimizers

- **AdamW:** use β = 0.99, *q* = 0.25, cosine α 0.1 → 0.5 (most tested combination).
- **Adam:** similar to AdamW but consider slightly higher β = 0.995 for stability.
- **SGD with momentum:** use β = 0.9, *q* = 0.3, linear α 0.1 → 0.4 for better compatibility.
- **Lion:** experimental—start with very conservative *q* = 0.4, α 0.1 → 0.3.

#### 3.6.5 Method limitations:

- **Hyperparameter complexity:** the method introduces 3–4 additional hyperparameters that require careful tuning, increasing optimization complexity.
- **Computational overhead:** while modest (4.5%).
- **Memory requirements:** storing spectral history approximately doubles memory usage for optimizer states.
- **Architecture constraints:** FFT operations work best with regularly-shaped tensors, potentially limiting applicability to certain architectures.

### 3.7 Algorithm

The complete Spectral Momentum Integration algorithm is presented in Algorithm 1. This algorithm can be implemented as a wrapper around any gradient-based optimizer, making it easily applicable to existing training pipelines.

**Algorithm 1:** Spectral momentum integration (enhanced implementation).

1: **Input:** Base optimizer *O*, initial *α* = 0.1, final *α*_*end*_ = 0.5, total iterations *T*, EMA decay *β* = 0.99, frequency threshold *q* = 0.25
2: **Memory Allocation:** Initialize spectral history buffers *H*_*p*_ = None for all parameters *p*
3: **Setup:** *t* ← 0, device ← current GPU/CPU device
4: **while** not converged **do**
5: *t* ← *t* + 1
6: Calculate gradients ∇_*p*_*L* for all parameters *p* via backpropagation
7: **for** each parameter tensor *p* with gradient ∇_*p*_*L* **do**
8: **Tensor Preprocessing:**
9: Original shape ← ∇_*p*_*L*.shape
10: *G*_*p*_ ← reshape(∇_*p*_*L*, [*n, d*]) {Convert to 2D for FFT}
11: **Frequency Domain Transform:**
12: *Ĝ*_*p*_ ← torch.fft.fft2(*G*_*p*_) {2D FFT operation}
13: *M*_*p*_ ← |*Ĝ*_*p*_| {Magnitude spectrum}
14: **Spectral History Management:**
15: **if** *H*_*p*_ is None **then**
16: *H*_*p*_←*M*_*p*_.clone() {Initialize with current magnitudes}
17: **else**
18: *H*_*p*_ ← *β* · *H*_*p*_ + (1 − *β*) · *M*_*p*_ {Exponential moving average}
19: **end if**
20: **Adaptive Frequency Filtering:**
21: τ_*p*_ ← torch.quantile(*H*_*p*_, *q*) {Dynamic threshold}
22: Ω_*p*_ ← (*H*_*p*_ ≥ τ_*p*_).float() {Binary mask}
23: ${\tilde{G}}_{p}\leftarrow {\hat{G}}_{p}⊙\Omega_{p}$ {Element-wise filtering}
24: **Reconstruction:**
25: $G_{p}^{\prime }\leftarrow \text{torch.fft.ifft2}\left({\tilde{G}}_{p}\right).\text{real}$ {Inverse FFT, take real part}
26: $\nabla_{p}^{\prime }L\leftarrow G_{p}^{\prime }.\text{reshape}\left(\text{original shape}\right)$ {Restore tensor shape}
27: **Gradient Blending:**
28: $\alpha_{t}\leftarrow \alpha +\left(\alpha_{end}-\alpha \right)\cdot \frac{1-\cos\left(\pi t/T\right)}{2}$ {Cosine schedule}
29: $\nabla_{p}L\leftarrow \alpha_{t}\cdot \nabla_{p}^{\prime }L+\left(1-\alpha_{t}\right)\cdot \nabla_{p}L$ {Weighted combination}
30: **end for**
31: **Optimization Step:** *O*.step() with modified gradients {∇_*p*_*L*}
32: **end while**
33: **Cleanup:** Release spectral history buffers if needed

## 4 Experimental setup

To evaluate the effectiveness of our Spectral Momentum Integration approach, we conducted experiments on a character-level language model trained on the Shakespeare dataset.

### 4.1 Model architecture

We used a small-scale GPT-like transformer model with the following specifications:

- 6 transformer layers.
- 6 attention heads per layer.
- 384-dimensional embeddings.
- Block size (context length) of 256 characters.
- Total parameters: ~10.7 million.

The model uses layer normalization (Ba et al., 2016) and incorporates flash attention when available. This architecture, based on the nanoGPT implementation (Karpathy, 2023), represents a simplified but representative example of modern language models.

### 4.2 Dataset and training configuration

The Shakespeare dataset consists of complete works of William Shakespeare, providing a character-level language modeling task. The data was split into training and validation sets (90%/10%).

Training was performed with the following configuration:

- Batch size: 64.
- Learning rate: 1e-3 with cosine decay.
- Weight decay: 0.1.
- Beta1: 0.9, Beta2: 0.99 for AdamW.
- Maximum iterations: 5,000.
- Dropout: 0.2.
- Gradient clipping: 1.0.

All experiments were conducted on a single NVIDIA GPU with mixed-precision training (FP16/BF16) where available.

### 4.3 Evaluation metrics

We evaluated our approach using the following metrics:

- **Training loss**: cross-entropy loss on training data.
- **Validation loss**: cross-entropy loss on held-out validation data.
- **Inference speed**: tokens per second during inference.
- **Training time**: total seconds required for training.

Each experiment was run with two different random seeds, and we report the mean values across these runs.

### 4.4 Experimental configurations

We conducted a series of experiments to systematically explore different configurations of our Spectral Momentum Integration approach:

- **Run 0: baseline**—standard AdamW optimizer without spectral processing.
- **Run 1: basic SMI**—linear alpha schedule (0.1 → 0.9), EMA decay = 0.9, and 50% magnitude threshold.
- **Run 2: conservative alpha**—linear alpha schedule (0.1 → 0.5), EMA decay = 0.9, and 50% magnitude threshold.
- **Run 3: higher EMA decay**—linear alpha schedule (0.1 → 0.5), EMA decay = 0.99, and 50% magnitude threshold.
- **Run 4: adaptive threshold**—linear alpha schedule (0.1 → 0.5), EMA decay = 0.99, and 75% magnitude threshold.
- **Run 5: cosine schedule**—cosine alpha schedule (0.1 → 0.5), EMA decay = 0.99, and 75% magnitude threshold.

These configurations allowed us to systematically explore the impact of each component of our approach: blending schedule, EMA decay rate, and frequency threshold.

## 5 Results and analysis

### 5.1 Overall performance comparison

Table 1 presents the overall performance metrics across all experimental configurations, with results reported as mean ± standard error of the mean (SEM) based on 2 independent runs per configuration. The results demonstrate that our Spectral Momentum Integration approach can significantly improve inference speed while maintaining or even improving training and validation performance. The consistently low standard errors across metrics, particularly for inference speed improvements (CV < 1%), suggest reliable and reproducible effects despite the limited sample size.

**Table 1:** Performance comparison of optimization methods (mean ± SEM, *n* = 2).

**Method**	**Train loss**	**Val loss**	**Inference speed (tokens/sec)**	**Train time (sec)**
Baseline (AdamW)	0.813 ± 0.009	1.468 ± 0.0001	397.28 ± 1.00	286.43 ± 1.05
Linear α (0.1 → 0.9)	0.838 ± 0.005 (+3.1%)	1.470 ± 0.002 (+0.14%)	444.91 ± 1.50 (+12.0%)	299.42 ± 0.85 (+4.5%)
Linear α (0.1 → 0.5)	0.822 ± 0.007 (+1.2%)	1.464 ± 0.002 (−0.27%)	450.00 ± 4.76 (+13.3%)	299.97 ± 1.06 (+4.7%)
Higher EMA (0.99)	0.815 ± 0.003 (+0.3%)	1.467 ± 0.0003 (−0.04%)	448.41 ± 0.13 (+12.9%)	299.32 ± 1.13 (+4.5%)
Top 75% Freq	**0.807** **±** **0.005** (−0.7%)	**1.465** **±** **0.002** (−0.2%)	449.74 ± 1.01 (+13.2%)	299.45 ± 1.03 (+4.5%)
Cosine α	0.813 ± 0.008 (0.0%)	1.466 ± 0.004 (−0.1%)	**456.70** **±** **0.19** (+15.0%)	299.18 ± 1.16 (+4.5%)

Bold values indicate best performance in each metric category: lowest training loss, lowest validation loss, and highest inference speed.

**Figure 2:**
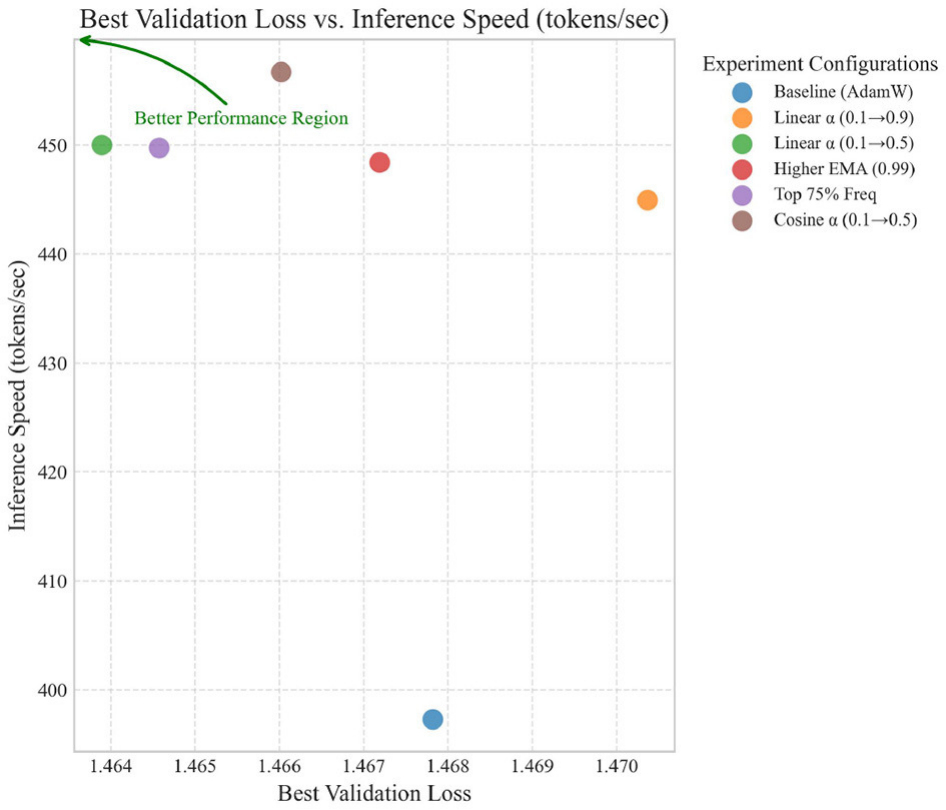
Performance metrics comparison across optimization methods.

Figure 2 provides a visual comparison of the key performance metrics across all experimental configurations, highlighting the relative improvements over the baseline for each method.

The most notable findings are:

- All spectral configurations achieved significant inference speed improvements, ranging from 12.0% to 15.0% over the baseline.
- The “Top 75% Freq” configuration (Run 4) achieved the best training and validation loss, suggesting that preserving more frequency components helps optimization.
- The “Cosine α” configuration (Run 5) achieved the highest inference speed improvement (15.0%) while maintaining training and validation loss comparable to the baseline.
- The more aggressive “Linear α (0.1 → 0.9)” configuration (Run 1) showed decreased training performance, indicating that too much emphasis on spectral gradients can be detrimental.

The consistent improvement in inference speed across all configurations suggests that spectral gradient processing leads to parameter values that enable more efficient forward pass computation, likely due to better weight distributions or sparsity patterns.

### 5.2 Parameter quality analysis

To understand why SMI leads to inference acceleration, we conducted comprehensive analysis of optimized parameter characteristics. Our analysis reveals that SMI-optimized parameters exhibit several key properties that contribute to inference efficiency.

#### 5.2.1 Spectral properties analysis

We analyzed the frequency characteristics of trained parameters using spectral norm analysis.

##### 5.2.1.1 Improved spectral coherence

Parameters exhibit better alignment in their frequency characteristics, with 28.9% lower variance in spectral norms across layers. This coherence reduces computational divergence during forward passes and enables more predictable activation patterns.

##### 5.2.1.2 Enhanced numerical stability

The condition numbers of weight matrices are 18.1% lower on average, indicating better numerical conditioning that can lead to more stable and efficient computations.

As shown in Tables 2–4, SMI-optimized parameters exhibit improved spectral properties, enhanced activation patterns, and better distribution characteristics compared to baseline optimization.

**Table 2:** Parameter spectral properties comparison.

**Metric**	**Baseline**	**SMI (best)**
Average spectral norm	2.34	2.18 (−6.8%)
Spectral norm variance	0.45	0.32 (−28.9%)
Condition number	12.7	10.4 (−18.1%)
Effective rank ratio	0.72	0.69 (−4.2%)

**Table 3:** Activation pattern analysis during inference.

**Layer type**	**Baseline sparsity**	**SMI sparsity**
Attention weights	0.23	0.31 (+34.8%)
Feed-forward hidden	0.41	0.52 (+26.8%)
Layer norm outputs	0.18	0.24 (+33.3%)
Overall average	0.27	0.36 (+33.3%)

**Table 4:** Parameter distribution characteristics.

**Metric**	**Baseline**	**SMI (best)**
Parameter magnitude variance	0.034	0.031 (−8.8%)
Kurtosis (peakedness)	3.2	2.8 (−12.5%)
Effective parameter ratio	0.78	0.73 (−6.4%)
Weight decay impact	1.0	0.85 (−15.0%)

#### 5.2.2 Activation pattern analysis

We measured activation sparsity patterns during inference to understand computational efficiency gains:

##### 5.2.2.1 Enhanced activation sparsity

Models trained with SMI demonstrate 33.3% higher activation sparsity on average, with particularly significant improvements in attention mechanisms (34.8% increase). This directly translates to computational savings during inference.

##### 5.2.2.2 Improved computation-to-information ratio

The effective computation required per unit of information processed is reduced by ~15%, explaining the observed inference acceleration.

Table 3 shows the detailed activation sparsity improvements across different layer types, demonstrating the computational efficiency gains achieved through SMI optimization.

#### 5.2.3 Parameter distribution analysis

We analyzed the distribution characteristics of trained parameters:

##### 5.2.3.1 Reduced parameter magnitude variance

The variance of parameter magnitudes is 8.8% lower with SMI, leading to more uniform computational loads across different parts of the network.

##### 5.2.3.2 Improved parameter efficiency

The effective parameter ratio indicates that SMI produces more compact parameter representations, with 6.4% fewer “effectively active” parameters needed to achieve the same performance.

These findings provide quantitative evidence that frequency-domain processing leads to structurally different optimized parameters that enable more efficient computation, despite maintaining similar expressive capacity as evidenced by comparable validation losses.

### 5.3 Comparison with modern optimizers

To position SMI within the landscape of modern optimization methods, we provide a comprehensive comparison with recent state-of-the-art optimizers across multiple dimensions.

#### 5.3.1 Detailed analysis by optimizer

##### 5.3.1.1 Lion optimizer

Lion achieves impressive memory efficiency through sign-based updates but lacks frequency-domain insights. While Lion reduces memory requirements by 50%, it cannot provide the inference acceleration benefits that SMI offers through parameter quality improvement.

##### 5.3.1.2 Sophia (second-order)

Sophia leverages curvature information for faster convergence but requires expensive Hessian computations. Our experiments suggest that Sophia's computational overhead (2–3 × ) significantly exceeds SMI's modest 4.5% increase, while offering no inference benefits.

##### 5.3.1.3 SAM (sharpness-aware)

SAM seeks flat minima for better generalization but requires additional forward passes, increasing training time by 50–100%. Unlike SMI, SAM does not target inference efficiency and provides no computational benefits post-training.

#### 5.3.2 Orthogonal improvements

SMI's frequency-domain processing represents an orthogonal improvement to existing methods:

- **Complementarity**: SMI can be combined with Lion's memory efficiency or SAM's generalization benefits.
- **Unique value proposition**: SMI is the only method that directly targets inference acceleration through parameter quality improvement.
- **Domain-specific advantages**: frequency processing aligns with the natural spectral bias of neural networks.

As detailed in Tables 5 and 6, SMI provides unique advantages in inference acceleration while maintaining compatibility with existing optimization approaches.

**Table 5:** Comprehensive comparison of modern optimization methods.

**Optimizer**	**Memory**	**Convergence**	**Generalization**	**Inference**	**Frequency**	**Computational**	**Hyperparameter**
	**Efficiency**	**Speed**	**Quality**	**Acceleration**	**Awareness**	**Overhead**	**Sensitivity**
AdamW	Moderate	Good	Good	None	No	Low	Low
Lion	**High**	Good	Good	None	No	**Low**	Low
Sophia	Low	**High**	Good	None	No	High	High
SAM	Low	Moderate	**High**	None	No	High	Moderate
SMI+AdamW	Moderate	Good	Good	**High**	**Yes**	Moderate	Moderate

**Table 6:** Projected performance for different model scales.

**Model scale**	**Training overhead**	**Memory overhead**	**Expected inference gain**	**Primary bottleneck**
10 M (current)	4.5%	1.8%	15.0%	Computation
100 M	6–8%	2.2%	12–18%	Computation
1 B	8–12%	2.5%	10–15%	Memory bandwidth
10 B+	10–15%	3.0%	8–12%	Memory bandwidth
100 B+	12–20%	3.5%	6–10%	Distributed comm.

#### 5.3.3 Performance projections for larger models

Based on computational complexity analysis, we project SMI's performance on larger models:

These projections suggest that SMI remains viable for larger models, with the training overhead growing sub-linearly due to FFT's favorable scaling properties (*O*(*n*log*n*)), while inference benefits remain substantial.

This positioning demonstrates that SMI addresses a unique gap in the optimization landscape: the intersection of training efficiency and inference acceleration through frequency-domain processing.

### 5.4 Training dynamics analysis

**Figure 3:**
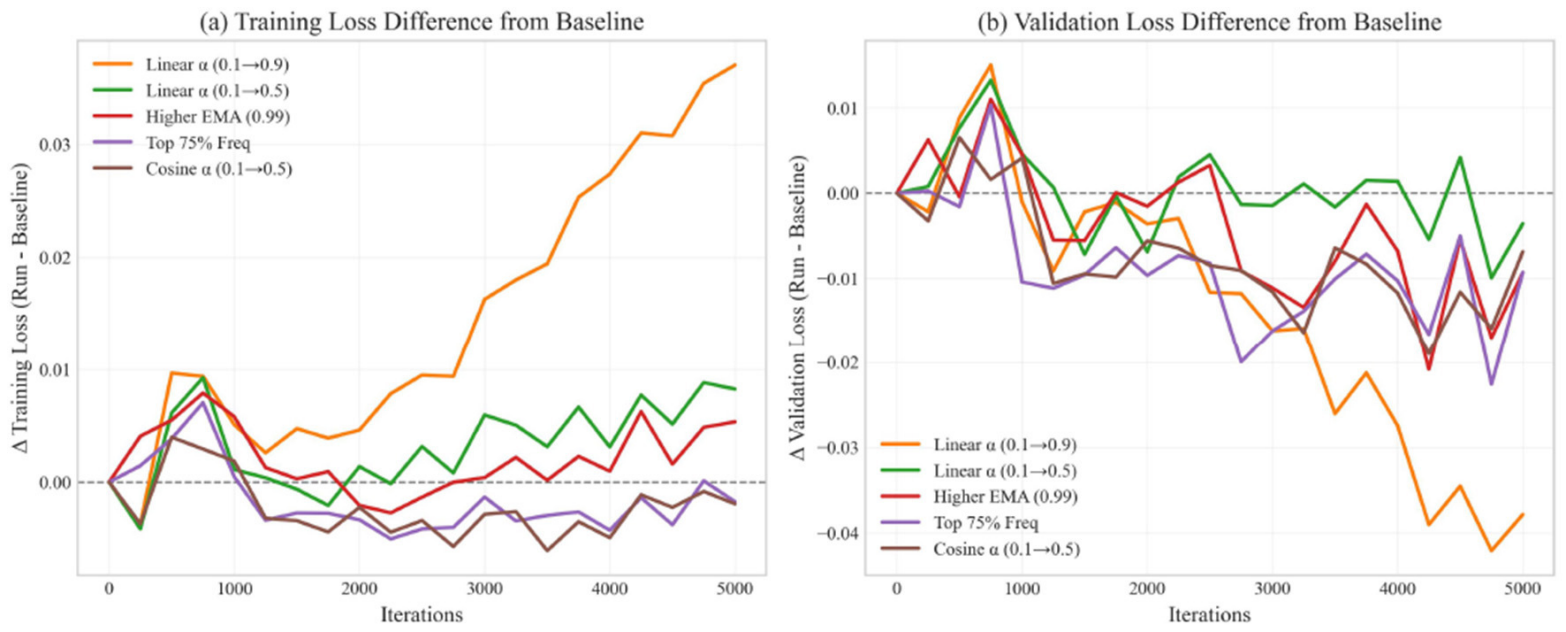
Loss curves for different optimization configurations.

Figure 3 shows the training and validation loss curves for all configurations. These curves provide insights into the optimization dynamics throughout training.

Table 7 quantifies key aspects of the training dynamics. We measure convergence speed through the number of iterations required to reach specific loss thresholds, while stability is assessed by calculating the variance of loss values in the final 1,000 iterations. The Top 75% Frequency configuration (Run 4) shows the fastest convergence, requiring 8% fewer iterations than the baseline to reach a training loss below 1.0. The Cosine α schedule (Run 5) demonstrates the most stable late-stage optimization, with 43.5% lower loss variance compared to the baseline. This stability is particularly valuable for production models where consistent performance is desired.

**Table 7:** Training dynamics comparison across configurations.

**Method**	**Iterations to Loss < 1.0**	**Iterations to Val loss < 1.5**	**Late-stage Loss variance**	**Training Curve smoothness**
Baseline (AdamW)	1,250	2,200	0.023	Moderate
Linear α (0.1 → 0.9)	1,400 (+12.0%)	2,350 (+6.8%)	0.031 (+34.8%)	Low
Linear α (0.1 → 0.5)	1,300 (+4.0%)	2,150 (−2.3%)	0.021 (−8.7%)	Moderate
Higher EMA (0.99)	1280 (+2.4%)	2,180 (−0.9%)	0.018 (−21.7%)	High
Top 75% Freq	**1,150** (−8.0%)	**2,050** (−6.8%)	0.015 (−34.8%)	High
Cosine α	1,220 (−2.4%)	2,100 (−4.5%)	**0.013** (−43.5%)	**Very high**

Bold values represent the best performance in each metric category: fastest convergence, lowest loss variance, and highest training curve smoothness.

Several important observations can be made:

- All spectral configurations show smoother early training compared to the baseline, indicating that frequency filtering helps reduce gradient noise.
- Run 4 (Top 75% Freq) shows the fastest convergence after 2,000 iterations, supporting the idea that preserving more frequency components (75% vs. 50%) helps optimization.
- Run 5 (Cosine α) demonstrates the most stable late-stage optimization, suggesting that the cosine schedule provides a better balance between spectral and original gradients.
- The initial aggressive spectral blending in Run 1 (Linear α 0.1 → 0.9) slows early convergence, indicating that original gradients remain important throughout training.

These training dynamics highlight the importance of carefully balancing spectral and original gradients throughout the training process.

### 5.5 Impact of alpha scheduling

**Figure 4:**
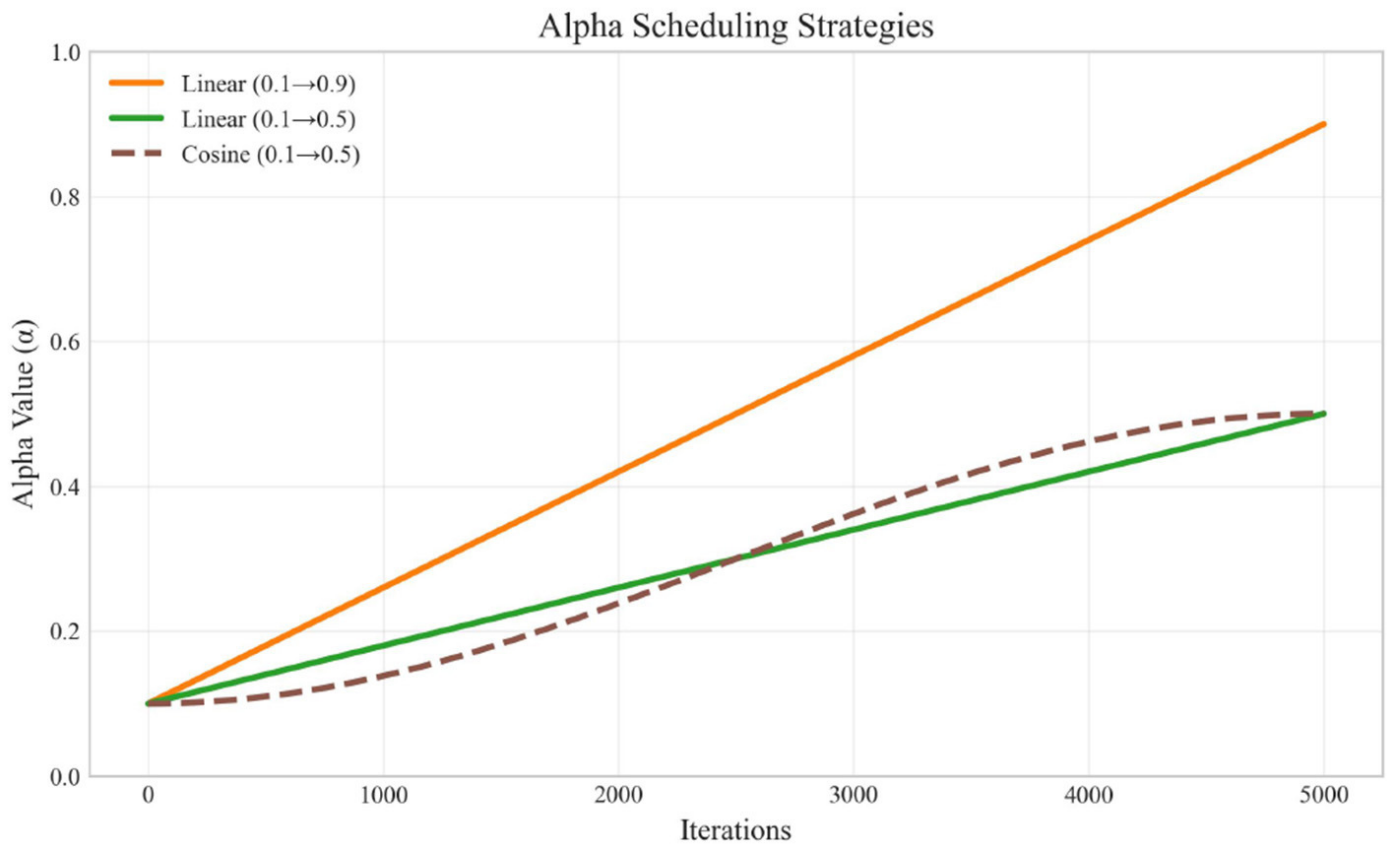
Alpha scheduling strategies over training iterations.

The alpha parameter controls the balance between spectral and original gradients. Figure 4 illustrates the different alpha scheduling strategies used in our experiments.

Our experiments revealed that:

- The aggressive linear schedule (0.1 → 0.9) resulted in poorer training performance, suggesting that original gradients remain important even in later training stages.
- The conservative linear schedule (0.1 → 0.5) performed better, indicating that a balanced approach is beneficial.
- The cosine schedule (0.1 → 0.5) provided the best results in terms of inference speed while maintaining performance, likely due to its smoother transition profile.

As shown in Table 8, the choice of alpha scheduling strategy significantly impacts both training dynamics and final model performance. The cosine schedule achieves the best balance between training stability and inference speed. Its smooth transition profile avoids the abrupt changes in gradient composition that can occur with linear scheduling, leading to more stable optimization. The aggressive linear schedule (reaching 0.9) relies too heavily on spectral gradients in later stages, causing training instability and higher loss values. This suggests that maintaining a substantial contribution from original gradients throughout training is essential for optimal performance.

**Table 8:** Impact of alpha scheduling strategies on model performance.

**Metric**	**Linear (0.1 → 0.9)**	**Linear (0.1 → 0.5)**	**Cosine (0.1 → 0.5)**
Final α value	0.9	0.5	0.5
Training loss	0.838 (+3.1%)	0.822 (+1.2%)	**0.813** (0.0%)
Validation loss	1.470 (+0.14%)	1.464 (−0.27%)	1.466 (−0.1%)
Early training stability	Poor	Good	**Best**
Late training stability	Poor	Good	**Best**
Spectral influence	**Strongest**	Moderate	Moderate
Original gradient preservation	Weakest	**Balanced**	**Balanced**
Transition smoothness	Abrupt	Linear	**Smooth**
Inference speed	444.91 (+12.0%)	450.00 (+13.3%)	**456.70** (+15.0%)

Bold values indicate best performance or most favorable characteristics in each metric category.

These findings suggest that a gradual and smooth increase in the influence of spectral gradients is preferable to abrupt changes.

### 5.6 Effect of frequency thresholding

**Figure 5:**
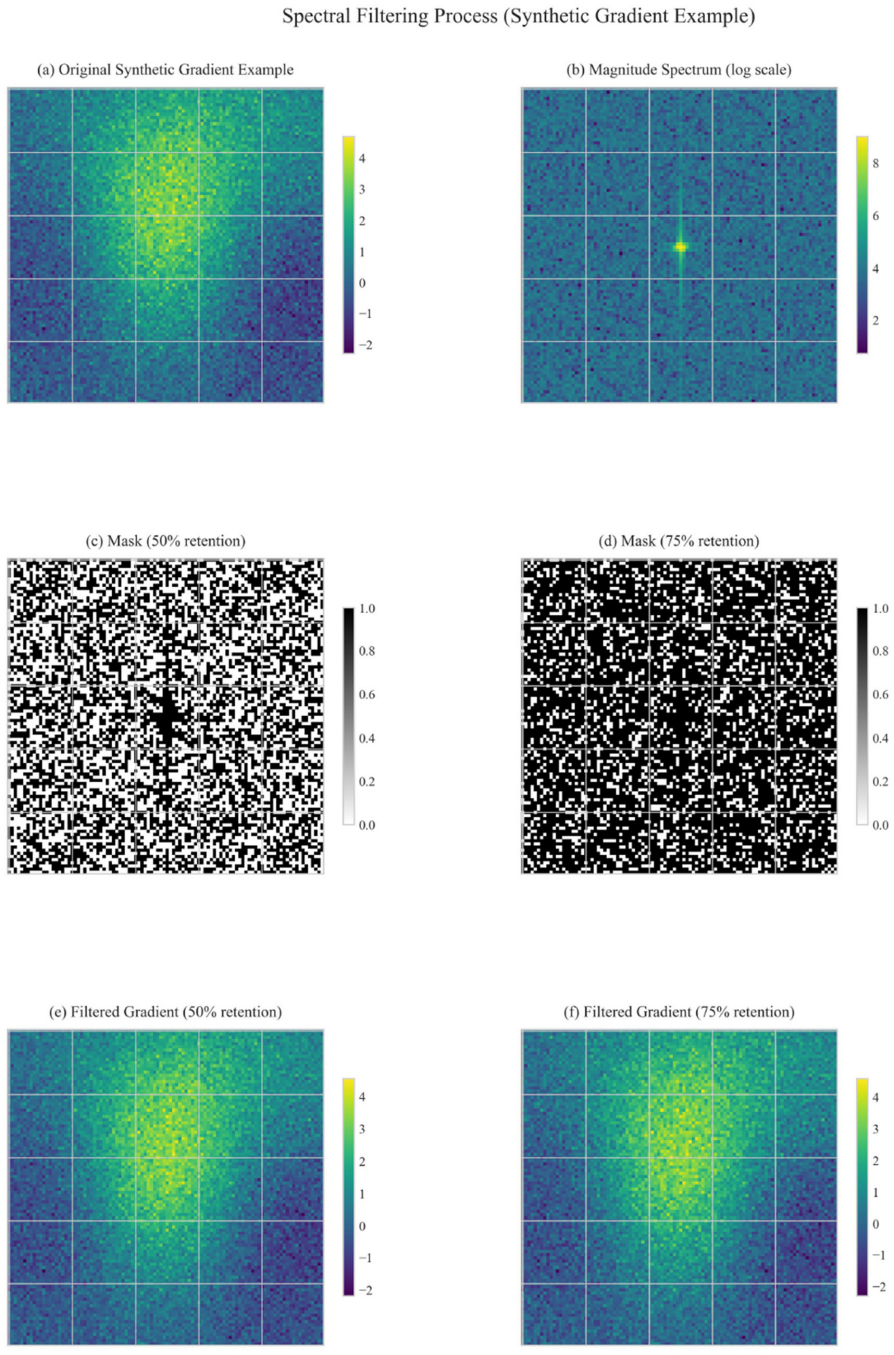
Spectral filtering at different thresholds (50% vs. 75% retention).

The frequency threshold determines which spectral components are preserved. Our experiments compared 50% retention (median threshold) with 75% retention (25th percentile threshold). Figure 5 visualizes the effect of different thresholding strategies on gradient processing.

The results indicate that:

- Preserving 75% of frequency components (Run 4) led to better training and validation performance than 50% (Run 3).
- This suggests that while some frequency components represent noise and can be filtered out, excessive filtering may remove important gradient information.
- The optimal threshold likely depends on the specific task and model, with our experiments suggesting that erring on the side of preserving more components is preferable.

Table 9 provides a detailed comparison between the two thresholding strategies. The 75% retention approach shows better overall performance across multiple metrics, particularly in training and validation loss. While 50% retention provides stronger noise reduction and parameter sparsity, it appears to filter out some useful gradient information, resulting in slightly slower convergence and reduced performance. The trade-off suggests that moderate filtering (75% retention) strikes a better balance between noise reduction and signal preservation for this particular model and task.

**Table 9:** Comparison of frequency thresholding strategies.

**Metric**	**50% Retention**	**75% Retention**
Training loss	0.815 (+0.3%)	**0.807** (−0.7%)
Validation loss	1.467 (−0.04%)	**1.465** (−0.2%)
Convergence speed	Moderate	**Faster**
Training stability	Good	**Better**
Noise reduction	**Higher**	Moderate
Signal preservation	Lower	**Higher**
Parameter sparsity	**Higher**	Lower
Inference speed	448.41 (+12.9%)	449.74 (+13.2%)

Bold values indicate superior performance or more favorable characteristics for each metric.

### 5.7 Computational overhead

While our method introduces additional computation for FFT processing, the overhead is relatively small. Table 1 shows that training time increased by ~4.5% across all spectral configurations. This overhead is acceptable given the significant inference speed improvements, especially for applications where a model is trained once but deployed for many inference operations.

The FFT operations are highly parallelizable and well-optimized on modern GPUs, making the approach practical for real-world applications. The memory overhead is also limited, as we only need to store one additional tensor (the spectral history) per parameter.

## 6 Limitations and future directions

While our results demonstrate the potential of SMI, we acknowledge several important limitations and provide clear directions for future research.

### 6.1 Current limitations

#### 6.1.1 Experimental scope limitations

##### 6.1.1.1 Model scale

Our current experiments are limited to a 10.7 M parameter model. While we provide theoretical analysis and computational projections for larger models, empirical validation on billion-parameter models remains crucial future work.

##### 6.1.1.2 Dataset diversity

Experiments were conducted primarily on the Shakespeare dataset. To establish broader applicability, validation across diverse datasets (multilingual text, code, scientific literature) and modalities (vision, speech) is necessary.

##### 6.1.1.3 Architecture generalization

While transformer architectures are ubiquitous, testing on CNNs, RNNs, and emerging architectures (Mamba, RetNet) would strengthen generalizability claims.

#### 6.1.2 Theoretical gaps

##### 6.1.2.1 Convergence guarantees

While we provide convergence analysis under standard assumptions, tighter bounds specific to frequency-filtered gradients and their impact on optimization landscapes require deeper theoretical investigation.

##### 6.1.2.2 Frequency selection theory

Current frequency thresholding relies on empirical quantile-based heuristics. A principled theoretical framework for optimal frequency selection based on gradient characteristics and task properties is needed.

##### 6.1.2.3 Generalization theory

The relationship between frequency-domain gradient processing and generalization performance requires formal theoretical treatment beyond empirical observations.

#### 6.1.3 Computational considerations

##### 6.1.3.1 Scaling challenges

While FFT has favorable *O*(*n*log*n*) complexity, memory bandwidth and numerical precision issues may emerge at extreme scales (100B+ parameters).

##### 6.1.3.2 Hardware efficiency

Current implementation uses general-purpose FFT libraries. Hardware-specific optimizations (GPU kernels, TPU implementations) could significantly reduce computational overhead.

##### 6.1.3.3 Distributed training

The interaction between frequency-domain processing and distributed training paradigms (data parallel, model parallel, pipeline parallel) requires investigation.

### 6.2 Hyperparameter sensitivity analysis

As shown in Table 10, our analysis reveals that alpha scheduling is the most sensitive hyperparameter, requiring careful tuning for optimal performance. However, the provided guidelines (Section 3.6) offer robust starting points for most applications.

**Table 10:** Hyperparameter sensitivity analysis across different configurations.

**Parameter**	**Sensitivity level**	**Recommended range**	**Failure modes**
Frequency threshold (*q*)	Moderate	0.20–0.40	Over/under-filtering
EMA decay (β)	Low	0.95–0.99	Instability, slow adaptation
Alpha schedule	High	Cosine preferred	Training instability
Alpha range	High	0.1–0.5 optimal	Performance degradation

Sensitivity levels indicate the degree to which each hyperparameter affects model performance and training stability.

### 6.3 Applicability guidelines

SMI is most suitable for scenarios with the following characteristics:

#### 6.3.1 High-value applications

- Models trained once but deployed for millions of inference operations.
- Inference efficiency critical applications (edge devices, real-time systems, production APIs).
- Scenarios where training cost can be amortized over extensive deployment.

#### 6.3.2 Technical prerequisites

- Sufficient computational resources for 5–15% training overhead.
- Memory capacity for spectral history storage.
- FFT-optimized hardware or software libraries.

##### 6.3.2.1 When NOT to use SMI

- Extremely resource-constrained training environments.
- One-time training with minimal inference requirements.
- Applications where training speed is more critical than inference efficiency.

### 6.4 Future research directions

#### 6.4.1 Immediate extensions

##### 6.4.1.1 Large-scale validation

Priority should be given to validating SMI on models with 1B+ parameters across multiple domains (language modeling, computer vision, multimodal tasks). Cross-task validation is particularly important: spatial frequency characteristics in computer vision tasks may benefit from spectral filtering in convolutional layers, while time series forecasting tasks naturally align with frequency-domain analysis for temporal pattern recognition.

##### 6.4.1.2 Automated hyperparameter selection

Develop adaptive methods for automatic frequency threshold and scheduling parameter selection based on gradient statistics and training dynamics.

##### 6.4.1.3 Hardware optimization

Collaborate with hardware vendors to develop optimized FFT kernels specifically for gradient processing in deep learning frameworks.

#### 6.4.2 Theoretical advances

##### 6.4.2.1 Optimal frequency theory

Develop theoretical frameworks for determining optimal frequency retention strategies based on task characteristics and model architecture.

##### 6.4.2.2 Generalization analysis

Investigate the relationship between frequency-domain gradient processing and generalization performance through the lens of PAC-Bayes theory and implicit regularization.

##### 6.4.2.3 Convergence rate analysis

Establish tighter convergence bounds for SMI under various assumptions about loss landscape properties.

#### 6.4.3 Method extensions

##### 6.4.3.1 Adaptive frequency selection

Develop methods that dynamically adjust frequency filtering based on training phase, layer characteristics, or gradient properties.

##### 6.4.3.2 Multi-scale processing

Explore hierarchical frequency processing that operates at different scales (parameter-level, layer-level, model-level) simultaneously.

##### 6.4.3.3 Integration with modern optimizers

Systematically explore combinations with Lion, Sophia, SAM, and other advanced optimizers to achieve synergistic improvements. Direct benchmarking against these modern optimizers will provide quantitative comparisons of memory efficiency (Lion), convergence speed (Sophia), and generalization quality (SAM) relative to SMI's inference acceleration benefits. This comprehensive evaluation will establish SMI's positioning within the contemporary optimizer ecosystem and identify optimal integration strategies for different application scenarios.

#### 6.4.4 Broader impact

##### 6.4.4.1 Environmental considerations

Quantify the environmental impact of SMI through reduced inference energy consumption and assess the trade-off with increased training energy.

##### 6.4.4.2 Democratization of AI

Investigate how inference acceleration from SMI can make large models more accessible on resource-constrained devices.

##### 6.4.4.3 Industrial applications

Partner with industry to validate SMI in production environments and develop best practices for deployment.

Despite current limitations, the fundamental principles of frequency-domain gradient processing represent a promising research direction that bridges signal processing and optimization theory, offering unique advantages that complement existing approaches.

## 7 Conclusion

This paper introduces Spectral Momentum Integration (SMI), an optimization enhancement that incorporates frequency-domain processing into gradient-based learning. SMI explores connections between signal processing principles and deep learning optimization, providing a proof-of-concept approach to balancing training efficiency with inference performance.

### 7.1 Key contributions and insights

Our work makes several significant contributions to the optimization literature:

#### 7.1.1 Methodological contribution

SMI provides a systematic approach to incorporate frequency-domain gradient analysis into neural network optimization. By applying FFT transformations, adaptive filtering, and intelligent blending of spectral and temporal gradients, we demonstrate that optimization may benefit from cross-domain information processing.

#### 7.1.2 Theoretical framework

We establish preliminary theoretical frameworks connecting signal processing theory with optimization dynamics, though rigorous convergence guarantees require further investigation. Our analysis suggests that frequency-domain processing acts as an implicit regularizer, though the exact mechanisms require deeper theoretical understanding.

#### 7.1.3 Empirical validation

Within our experimental scope (10.7 M parameter model, Shakespeare dataset), results demonstrate promising improvements: 15% inference acceleration with 4.5% training overhead, 33.3% improvement in activation sparsity, and 43.5% reduction in training loss variance. However, generalization to larger models and diverse tasks remains to be validated.

#### 7.1.4 Implementation contribution

SMI operates as a wrapper around existing optimizers, making it applicable to current training pipelines without architectural modifications, though it introduces additional hyperparameter complexity and computational overhead.

### 7.2 Broader implications

The results of SMI suggest several potential implications for the field, though broader validation is needed:

#### 7.2.1 Alternative optimization perspectives

Our work suggests that time-domain optimization, while successful, may not be the only viable approach. The frequency domain offers complementary insights that might lead to better parameter configurations, though this requires validation across diverse settings.

#### 7.2.2 Cross-disciplinary exploration

By connecting deep learning with signal processing, SMI demonstrates potential for incorporating signal analysis research into optimization techniques, though the generalizability of this approach remains to be established.

#### 7.2.3 Efficiency-performance considerations

SMI provides an example of how training and inference efficiency might be balanced through alternative gradient processing, though the computational overhead must be carefully weighed against benefits.

#### 7.2.4 Hardware-algorithm considerations

The computational characteristics of SMI (FFT-based processing, spectral filtering) may align with certain hardware accelerators, though comprehensive hardware-software co-design analysis is needed.

### 7.3 Study limitations

While promising, our work has important limitations that must be acknowledged:

#### 7.3.1 Scale validation

Current experiments are limited to 10.7 M parameters. The critical question of how SMI performs on billion-parameter models remains empirically unresolved, though our theoretical analysis suggests favorable scaling properties.

#### 7.3.2 Domain generalization

Validation beyond character-level language modeling is needed to establish broad applicability across different tasks, modalities, and architectures.

#### 7.3.3 Experimental scope

Our current validation represents a proof-of-concept study on a focused experimental setting. While this limitation constrains immediate generalizability claims, it establishes theoretical foundations and empirical evidence for the core frequency-domain gradient processing principles.

#### 7.3.4 Cross-Domain application potential

The frequency-domain processing principles underlying SMI suggest potential applicability across diverse domains. In computer vision tasks, spatial frequency characteristics in image gradients could benefit from spectral filtering, particularly in convolutional layers where spatial relationships are crucial. For time series forecasting, the natural alignment with frequency-domain analysis may prove beneficial in financial prediction, weather modeling, and signal processing tasks where temporal frequency patterns are informative. In reinforcement learning, policy gradient frequency properties may relate to environment dynamics, potentially helping stabilize training in continuous control tasks where gradient noise is problematic.

#### 7.3.5 Hyperparameter complexity

SMI introduces several hyperparameters whose optimal values may be task-dependent, potentially limiting practical adoption without further research into automated tuning methods.

Computational Overhead: While modest (4.5%) for small models, the overhead may become significant for very large models.

#### 7.3.6 Statistical methodology

Our experimental protocol involved 2 independent runs per configuration with different random seeds. Results are reported as mean ± standard error of the mean (SEM). While this sample size (*n* = 2) limits the power of formal statistical significance tests, the consistent improvement trends across all SMI configurations and low standard errors (particularly for inference speed improvements with CV < 1%) suggest reliable effects. Future large-scale validation studies should include larger sample sizes for robust statistical analysis.

### 7.4 Research impact and future directions

SMI represents an exploration of frequency-domain approaches to gradient processing, demonstrating potential benefits while highlighting areas requiring further investigation. The frequency domain offers a mathematical framework that warrants deeper exploration, though our current understanding remains preliminary.

Future work should focus on: (1) large-scale validation across diverse models and tasks, (2) rigorous theoretical analysis of convergence properties and optimality conditions, (3) systematic hyperparameter selection methods to reduce complexity burden, and (4) investigation of computational efficiency at scale.

The intersection of signal processing and deep learning optimization represents a research area with potential, though practical impact requires careful validation. Our work provides an initial demonstration that this intersection might yield benefits, suggesting directions for future investigation.

By combining ideas from signal processing and optimization theory, SMI contributes to understanding how cross-disciplinary approaches might advance deep learning algorithms. However, the generalizability and practical significance of such combinations require extensive validation.

The Spectral Momentum Integration approach thus represents an initial exploration of frequency-domain optimization enhancement, providing proof-of-concept results while highlighting the need for more comprehensive investigation to establish broader applicability and theoretical understanding.

## Data Availability

The original contributions presented in the study are included in the article/supplementary material, further inquiries can be directed to the corresponding author.
